# Nanoengineered Shear-Thinning Hydrogel Barrier for Preventing Postoperative Abdominal Adhesions

**DOI:** 10.1007/s40820-021-00712-5

**Published:** 2021-10-18

**Authors:** Guillermo U. Ruiz-Esparza, Xichi Wang, Xingcai Zhang, Sofia Jimenez-Vazquez, Liliana Diaz-Gomez, Anne-Marie Lavoie, Samson Afewerki, Andres A. Fuentes-Baldemar, Roberto Parra-Saldivar, Nan Jiang, Nasim Annabi, Bahram Saleh, Ali K. Yetisen, Amir Sheikhi, Thomas H. Jozefiak, Su Ryon Shin, Nianguo Dong, Ali Khademhosseini

**Affiliations:** 1 Biomaterials Innovation Research Center, Division of Engineering in Medicine, Department of Medicine, Brigham and Women’s Hospital, Harvard Medical School, Cambridge, MA 02139 USA; 2 Division of Health Sciences and Technology, Harvard University – Massachusetts Institute of Technology, Cambridge, MA 02139 USA; 3 Department of Cardiovascular Surgery, Union Hospital, Tongji Medical College, Huazhong University of Science and Technology, Wuhan, 430022 People’s Republic of China; 4 School of Engineering and Applied Sciences, Harvard University, Cambridge, MA 02138 USA; 5 School of Engineering and Science, Tecnologico de Monterrey, Campus Monterrey, Monterrey, Nuevo Leon 64849 Mexico; 6 School of Medicine and Health Science, Campus Guadalajara, Zapopan, Jalisco 45201 Mexico; 7 West China School of Basic Medical Sciences and Forensic Medicine, Sichuan University, Chengdu, 610041 China; 8 Department of Chemical and Biomolecular Engineering, University of California, Los Angeles, Los Angeles, CA 90095 USA; 9 Department of Chemical Engineering, Northeastern University, Boston, MA 02115 USA; 10 Department of Chemical Engineering, Imperial College London, London, SW7 2AZ UK; 11 Department of Chemical Engineering, The Pennsylvania State University, University Park, PA 16802 USA; 12 Department of Biomedical Engineering, The Pennsylvania State University, University Park, PA 16802 USA; 13 Terasaki Institute for Biomedical Innovation, 11570 W Olympic Blvd, Los Angeles, CA 90024 USA

**Keywords:** Postoperative adhesions, Shear-thinning hydrogel, Silicate nanoplatelets, Nanotechnology, Nanomedicine

## Abstract

More than 90% of surgical patients develop postoperative adhesions, and the incidence of hospital re-admissions can be as high as 20%. Current adhesion barriers present limited efficacy due to difficulties in application and incompatibility with minimally invasive interventions. To solve this clinical limitation, we developed an injectable and sprayable shear-thinning hydrogel barrier (STHB) composed of silicate nanoplatelets and poly(ethylene oxide). We optimized this technology to recover mechanical integrity after stress, enabling its delivery though injectable and sprayable methods. We also demonstrated limited cell adhesion and cytotoxicity to STHB compositions in vitro. The STHB was then tested in a rodent model of peritoneal injury to determine its efficacy preventing the formation of postoperative adhesions. After two weeks, the peritoneal adhesion index was used as a scoring method to determine the formation of postoperative adhesions, and STHB formulations presented superior efficacy compared to a commercially available adhesion barrier. Histological and immunohistochemical examination showed reduced adhesion formation and minimal immune infiltration in STHB formulations. Our technology demonstrated increased efficacy, ease of use in complex anatomies, and compatibility with different delivery methods, providing a robust universal platform to prevent postoperative adhesions in a wide range of surgical interventions.

**Supplementary Information:**

The online version contains supplementary material available at 10.1007/s40820-021-00712-5.

## Highlights

- A novel “nanoengineered hydrogel” barrier based on silicate nanoplatelets and poly(ethylene oxide) (PEO) was developed to prevent the formation of postoperative adhesions.
- Compared to other hydrogel systems, the prepared biomaterial is injectable and sprayable which makes it compatible with minimally invasive interventions.

**Supplementary Information:**

The online version contains supplementary material available at 10.1007/s40820-021-00712-5.

## Graphical abstract

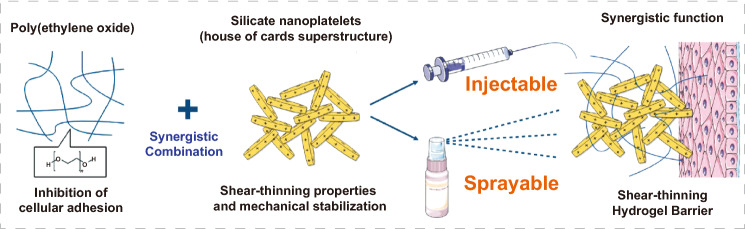

## Introduction

One of the most challenging medical problems associated with surgical interventions has been the formation of adhesions, as 93% of patients who undergo open pelvic or abdominal surgery develop this pathology [1]. Postoperative adhesions are pathologic formations of fibrotic tissue that occur after peritoneal injury and adhere the inner peritoneal lining of the abdominopelvic wall to internal organs or tissues within the abdominal or pelvic cavities (intestines, liver, gallbladder, urinary bladder, uterus, fallopian tubes, ovaries) [2]. A multifactorial cascade that involves ischemia, inflammation, angiogenesis, and tissue repair is known to cause their formation [3]. Adhesions are associated with a significant decrease in quality of life, morbidity and mortality, and the incidence of hospital re-admissions due to their complications (intestinal obstruction, chronic abdominopelvic pain, and secondary infertility) is as high as 20% [1, 4]. The catastrophic financial costs for the health care system are ~ $1.3 billion in the USA alone, and in Europe, costs are higher than the surgical expenditure for gastric and rectal cancers [5].

Physical barriers in the form of films are commonly used to prevent adhesions by avoiding direct contact of injured and uninjured serosal surfaces prone to adhesion formation during the healing phase, however, their application to irregular surfaces and cavities is challenging or impossible, since they are fragile, difficult to handle, incompatible with minimally invasive laparoscopic or catheter-based procedures, and their limited efficacy (~ 25%) decreases their clinical adoption. Therefore, the development of novel technologies to solve these clinical limitations is urgently needed.

Hydrogel formulations for the prevention of postoperative adhesions may be especially attractive as a substitute for commercially available barriers. The ideal biomaterial requires unique mechanical and biological properties to prevent cell adherence, infiltration, and adhesion formation [6–8]. The technology must be injectable to be compatible with minimally invasive procedures, such as laparoscopies and arthroscopies, and sprayable to uniformly cover large and irregular areas during laparotomies or thoracotomies [3], a limitation that current ‘film-based’ barriers have.

**Fig. 1:**
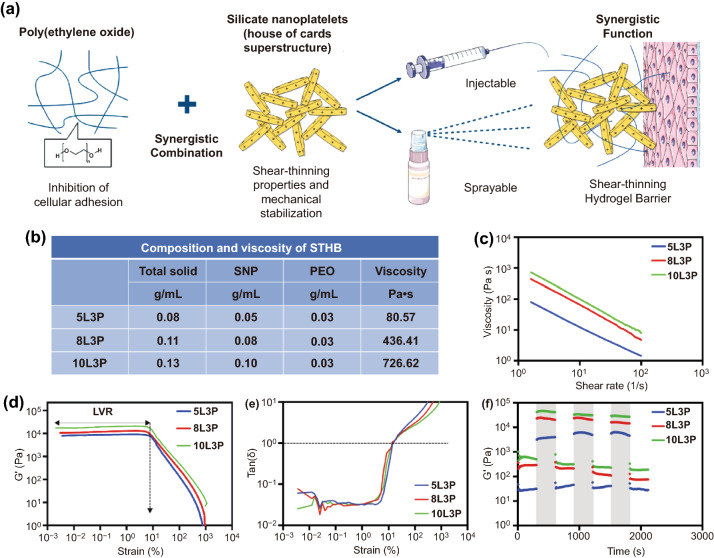
STHB formulation and rheological characterization. **a** Schematic representation of STHB formulation and delivery methods. **b** The composition and viscosity of STHB formulations is presented in several SNP/PEO ratios. Inherent viscosity was obtained by recording the maximum value during rheological shear rate sweeps after a 5-min equilibration at 37 °C. **c** Viscosity versus shear rate was obtained, viscosity decreased as shear rate increased, illustrating the shear-thinning properties of the compositions. **d** Strain (0.001 to 1000% at 1 Hz) versus storage moduli (G′) was quantified to determine the linear viscoelastic regions (LVR) of STHB formulations, the plateau of G’ indicates the strain resistance of the compositions before deformation and transition to a liquid-like state. **e** Tan (*δ*) versus strain was calculated to determine the elastic to viscous transformation, the gel point [tan(*δ*) = 1] was found at ~ 10 strain (%). **f** Storage moduli (G′) was recorded during multiple cycles of low (1%) and high (100%) strain, the light gray regions indicate rapid sample recovery to its original modulus

To achieve the desired mechanical and biological properties, a hydrogel composed of silicate nanoplatelets (SNP) and poly(ethylene oxide) (PEO) was nanoengineered. The disk-shaped SNPs (thickness =  ~ 0.92 nm and diameter =  ~ 25 nm) possess unique electrostatic properties with negatively charged surfaces and positively charged edges, that result in a nanoscale surface-to-edge attraction and spontaneous formation of a superstructure, that enables its self-assembly and gelation upon water dissolution. The dual electrostatic charges of SNPs confer non-Newtonian and shear-thinning behavior to the system, allowing its injectability and sprayability after subjecting the material to stress, with subsequent mechanical recovery immediately after delivery (Fig. 1a) [9]. Additionally, the unique self-assembly properties of SNPs allow them to form a nanostructured ultra-efficient barrier, providing an organized tortuous network that prevents and reduces the transport of molecules and colloidal particles [10]. To complement this system, PEO (M.W. 20,000), a biocompatible polymer was carefully selected, as its molecular weight provides unique biological antifouling properties, low immunogenicity and minimal binding sites for cell adherence or protein adsorption, ultimately preventing cellular infiltration and growth [11].

It is hypothesized that the rational design and synergistic combination of the mechanical and biological features of the SNPs and PEO provide a unique and versatile shear-thinning hydrogel barrier (STHB) that can be applied via multiple facile delivery methods. This technology is designed to provide physical separation between tissues and inhibit infiltration of collagen-secreting cells, offering a universal solution to prevent the formation of postoperative adhesions in a wide range of surgical procedures without technical limitations.

## Experimental Section

### STHB Formulation

STHBs were formulated with different percentages of poly(ethylene oxide) (PEO) (M.W. 20,000) (Sigma-Aldrich, St. Louis, MO) and silicate nanoplatelets (SNPs) (Laponite XLG) (BYK, Wesel, Germany). PEO and SNPs were sterilized using UV light and dissolved in ultra-filtered deionized water in separate vials. For dissolution, SNPs were stirred (400 rpm) at 60 °C, and after 1 min of stirring, PEO solution was added before gelation and stirred for four additional minutes until complete homogenization was achieved. The hydrogel compositions that were fabricated are 5 wt% SNP (5L), 8 wt% SNP (8L), 10 wt% SNP (10L), 5 wt% SNP 1 wt% PEO (5L1P), 8 wt% SNP 1 wt% PEO (8L1P), 10 wt% SNP 1 wt% PEO (10L1P), 5 wt% SNP 2 wt% PEO (5L2P), 8 wt% SNP 2 wt% PEO (8L2P), 10 wt% SNP 2 wt% PEO (10L2P), 5 wt% SNP 3 wt% PEO (5L3P), 8 wt% SNP 3 wt% PEO (8L3P), and 10 wt% SNP 3 wt% PEO (10L3P). Clear hydrogel solutions were obtained and stored at room temperature for 48 h to achieve stabilization.

### Cell Line and Cell Culture Supplies

3T3 fibroblasts and THP-1 cells (ATCC, Manassas, VA) were cultured in Dulbecco’s modified eagle medium (DMEM) (Invitrogen, Carlsbad, CA) supplemented with 10% fetal bovine serum (FBS) (Atlanta Biologicals, Flowery Branch, GA) and 1% penicillin–streptomycin (PS) (MediaTech Inc., Manassas, VA) under 5% CO_2_ at 37 °C. Cells were passaged approximately two times per week, and media was exchanged every 2 days.

### Rheological Characterization of STHB Formulations

To characterize the rheological and mechanical properties of STHB formulations, a MCR 301 rheometer was used (Anton Paar, Graz, Austria) as previously described [9]. Temperature sweeps were performed on a 25-mm-diameter plate (gap height: 500 μm), and mineral oil was placed around to prevent water evaporation. Equilibration time was set to 10 min before testing, followed by steady shear at 10 s^−1^ for 2 min. Shear rate sweeps (0 to 100 s^−1^ with 10 points/decade) and strain sweeps (0.001 to 1000% at 1 Hz) were performed at 37 °C. Recovery testing was performed by applying a value outside of the linear viscoelastic range (100% strain), followed by a value inside of the linear viscoelastic range (1% strain) at 1 Hz.

### Injection Force Test

The injection force required to extrude STHB formulations was analyzed using a mechanical tester (Instron Model 5542) (Instron, Norwood, MA). STHB formulations were loaded into 3-mL syringes (BD Biosciences, San Jose, CA) and injected through three different intraluminal diameter needle sizes, 0.838 mm (18G), 0.337 mm (23G), and 0.210 mm (27G) (BD Biosciences, San Jose, CA). The syringe plungers were pressed by an upper compressive platen, and the lower housing of the syringe was placed into the tensile grip of the instrument to prevent movement. The injection rate used was 2 mL min^−1^, and the force on the plunger was measured with a 100-N load cell. All STHB samples were tested in triplicate. Bluehill version 3 software (Instron, Norwood, MA) was used to analyze the data.

### Sprayability of STHB Formulations

To determine the sprayability of STHB formulations and facilitate their visualization, hydrogel compositions were labeled with Alexa Fluor 488 dye (Thermo Fisher Scientific, Waltham, MA). STHB formulations were loaded into 10 mL syringes (BD, Franklin Lakes, NJ) and sprayed through a specialized setup. The setup consisted on an infusion pump (Harvard Apparatus, Holliston, MA) to control the hydrogel extrusion rate, and a STHB-loaded syringe with dual nozzles, one connected to a pressurized nitrogen tank and a second one used to spray the hydrogel compositions. The parameters used to spray STHB formulations were: 100 kPa to infuse the hydrogels, 1 mL min^−1^ infusion rate, a 20 cm spraying distance, and a 22G nozzle with 0.41 mm of intraluminal diameter. To determine spray angle and area, photographs and videos were taken during the spraying of 5 mL of the compositions from a 20 cm distance. Spray angle was calculated by using the following formula: tangent (*x*) = opposite / adjacent. To determine the average spot area of the formulations, 0.1 mL of the compositions was sprayed from a 20 cm distance and videos and photographs were taken. Spray patterns were measured by performing area fraction analysis using ImageJ software (National Institutes of Health, Bethesda, MD) as previously reported [12].

### Spreadability Test

The spreadability of the hydrogels was evaluated 48 h after their preparation. STHB formulations were incubated for 1 h at 37 °C, and after incubation, the studies were quickly performed at room temperature. STHBs were placed between two horizontal transparent glass plates (Bio-Rad, Hercules, CA), and a 125 g weight was placed on the upper plate. After one minute, the weight was removed, and the spreading diameter was measured. The spreadability was quantified as the total diameter covered by the hydrogel within the plates, and its fluidity and stiffness classified according to a previously published work [13].

### Environmental Scanning Electron Microscopy

To determine the cohesiveness and morphology of the hydrogel system in hydrated form, an environmental A FEI/Philips XL30 FEG scanning electron microcopy system was used (FEI/Phillips, Hillsboro, OR). The sample was mounted on a microscopy holder with a base of carbon tape and imaged.

### STHB Stability and Swelling Test

To determine the stability and swelling ratio of STHB formulations, one gram of each formulation was placed in a cell strainer (Corning, Corning, NY) (*n* = 3). Each strainer was submerged in 7 mL of PBS on 6-well plates (Corning, Corning, NY) and incubated at 37 °C. Stability and swelling were recorded at 3, 7, 14, and 21 days by quantifying the wet weight and dry weight after lyophilization of STHB compositions. Degradation kinetics of the hydrogel formulations were calculated with the formula: mass loss percentage = (*M*_0_ – *M*_*d*_) / *M*_0_ × 100% [14]. *M*_0_ represents the original dry mass of the hydrogel, and *M*_*d*_ is the mass of the hydrogel in the dry state after PBS incubation. Swelling ratio was calculated using the following formula: swelling ratio (*Q*) = (*W*_*s*_ – *W*_*d*_) / W_d_ [14]. *W*_*s*_ represents the mass of the hydrogel after PBS incubation, and *W*_*d*_ is the original mass of the hydrogel.

### 3T3 Cellular Adherence Assay to STHB Formulas

To determine cellular adherence to the hydrogel compositions, 10 × 10^4^ 3T3 fibroblasts were cultured in 24-well culture plates (Corning Inc., Corning, NY) coated with 0.2 mL of each hydrogel formulation using an injection method to achieve a 2 mm thickness. Twenty-four hours after incubation, cells were washed with PBS to remove unattached cells. Remaining cells were detached with trypsin (Sigma-Aldrich, Darmstadt, Germany) for posterior quantification. Cell numbers were determined by PrestoBlue Cell Viability Reagent (Thermo Fisher Scientific, Waltham, MA) via a microplate reader (BioTek Synergy 2, Winooski, VT). Analysis was performed by using BioTek Gen5 software (BioTek Synergy 2, Winooski, VT).

#### Single-Cell Analysis of 3T3 Cells

Cellular morphology was evaluated after seeding 10 × 10^4^ 3T3 fibroblast on the surface of STHB formulations placed on 6-well culture plates (Corning Inc., Corning, NY). After being incubated for 24 h, cells were fixed using a 4% paraformaldehyde solution (Sigma-Aldrich, St. Louis, MO), followed by F-actin staining using phalloidin red (Thermo Fisher Scientific, Waltham, MA). Cellular fluorescence micrographs at different locations of the material surfaces were obtained using a fluorescence microscope (Zeiss, Oberkochen, Germany) and analyzed by Snap 2058-Zen Pro 2012 software (Zeiss, Oberkochen, Germany). Sixty individual cells per group were randomly selected in each micrograph for analysis. The maximum orthogonal length, width, and area of each cell were measured using ImageJ software (National Institutes of Health, Bethesda, MD), and the aspect ratio was calculated dividing the longer length by the shorter length of the cell.

#### THP-1 Cellular Adherence Assay to STHB Formulas

THP-1 cells were pre-treated for 24 h with TNF-α to induce an inflammatory phenotype, another group of cells remained untreated for the purpose of this experiment. After treatment, THP-1 cells (10 × 10^4^) were cultured in 24-well plates (Corning Inc., Corning, NY) coated with 0.2 mL of each STHB formulation and Matrigel Matrix (Corning, Inc., Corning, NY) as a positive control. After 24 h of incubation, cells were washed with PBS to remove unattached cells, F-actin was stained using phalloidin red (Thermo Fisher Scientific, Waltham, MA) and the nucleus using DAPI (Sigma-Aldrich, St. Louis, MO). Fluorescence microscopy images (Zeiss, Oberkochen, Germany) were taken to analyze cell adherence and quantify numbers per mm^2^ by Snap 2058-Zen Pro 2012 software (Zeiss, Oberkochen, Germany).

#### Cell Viability Test

Cytotoxicity of STHB formulations was evaluated, 10 × 10^3^ 3T3 fibroblasts were seeded in 96-well culture plates (Corning Inc., Corning, NY) and incubated for 48 h with the following ranges of SNPs, PEO and SNPs combined with PEO: 0.001 to 1000 μg mL^−1^. After the incubation period, cell viability was quantified by PrestoBlue Cell Viability Reagent (Thermo Fisher Scientific, Waltham, MA) via a microplate reader (BioTek Synergy 2, Winooski, VT) and BioTek Gen5 software (BioTek Synergy 2, Winooski, VT). A non-treated 3T3 fibroblasts control group was used to normalize the data of the treated groups and calculate the cell viability percentage.

#### Peritoneal Injury Model

The efficacy of STHB formulations to prevent the formation of postoperative adhesions was evaluated on 300 g male Wistar rats (Charles River Laboratories, Worcester, MA). Based on the in vitro results, the three most efficient STHB formulations (5L3P, 8L3P and 10L3P) were selected for in vivo testing, and compared to Seprafilm® (Sanofi, Paris, France) and a control group without any treatment or medical device. In total, five groups were tested, each group was composed of five animals. All animal experiments were conducted according to the NIH Guidelines for the Care and Use of Laboratory Animals. Protocol was approved by the Institutional Animal Care and Use Committee of Brigham and Women’s Hospital (2016N000472). After aseptic animal preparation, inhalable isoflurane was used as anesthesia. A laparotomy was performed using a standard midline incision of 4 cm, and eight peritoneal ischemic buttons were created using a chain distribution in parietal peritoneum (4 per side) to produce a peritoneal injury as previously described [15]. Each button was generated by using a Z-stitch technique with 2–0 polypropylene sutures (Ethicon, Somerville, NJ) to ligate 5 mm of peritoneal tissue. Afterwards, 0.1 mL of STHB (extruded through a syringe) or Seprafilm®, were applied on the surface of the ischemic buttons. STHB efficiently adhered to the tissues creating a homogenous coating in the ischemic buttons. Finally, the peritoneum of the abdominal incision was closed, and the skin layers were sutured separately. Analgesia was administered in the form of carprofen and buprenorphine during the first 48 h after surgery. Animals were kept alive for 14 days before adhesion formation was assessed.

#### Determination and Grading of Postoperative Adhesions

After 14 days, animals were euthanized according to the institutional guidelines and the severity of the adhesions was assessed by using the peritoneal adhesion index (PAI). The severity of adhesions was graded with the following scoring system: 0—no adhesion, 1—filmy adhesion that needs blunt dissection, 2—strong adhesion that needs sharp dissection, 3—very strong vascularized adhesion that needs sharp dissection with damage hardly preventable. Each button (8) was individually graded, and the index was calculated based on the sum of the total score of the eight buttons. Percentage of adhesion formation and efficacy of adhesion prevention were calculated based on the number of adhesions formed, and each button was considered as 12.5% of the total number of injuries created (eight ischemic buttons).

#### Histology and Immunohistology

After 14 days, tissues with peritoneal buttons were extracted, frozen, and sectioned into 7 μm sagittal and transversal slices using a HM550 Cryostat system (Thermo Fischer Scientific, Waltham, MA). Sections were stained with Hematoxylin & Eosin (Sigma-Aldrich, St. Louis, MO) and Masson’s trichrome (Sigma-Aldrich, St. Louis, MO) to assess tissue morphological changes and fibrotic formation. Anti-CD68 (Abcam, Cambridge, MA) and anti-CD3 (Abcam, Cambridge, MA) primary antibodies with Alexa Fluor–conjugated (Invitrogen, Carlsbad, CA) secondary antibodies were used in conjunction with DAPI (Vector Laboratories, Burlingame, CA) to perform immunohistology. Slides were examined (*n* = 5 pictures per section) using an Axio Observer microscope (Zeiss, Oberkochen, Germany) and a fluorescence microscope (Zeiss, Oberkochen, Germany).

#### Statistical Analysis

All the results are expressed as mean ± SD. An unpaired Student’s t test was used to determine statistical significance of all samples and groups. *P < 0.05, **P < 0.01 was considered statistically significant.

## Results and Discussion

### STHB Presents Rapid Modulus Recovery after Applied Strain

Biomaterials with shear-thinning properties are essential for the development of viscoelastic gel coatings that transform their mechanical properties to liquid-like states upon delivery under shear stress. This property enables their injectability or sprayability as a flowable liquid, and subsequent recovery to their original viscoelastic solid state when shear is removed after delivery. Previous attempts to provide sprayable hydrogel coatings as adhesion barriers have employed in situ chemistry (polymerization or cross-linking) to achieve the desired viscoelastic properties of hydrogel coatings delivered from a liquid precursor formulation. The STHB properties described in this study fundamentally enable the development of injectable and sprayable hydrogel compositions for standard and minimally invasive medical interventions without the need of additional polymerization or cross-linking [9, 16]. STHB technology achieves these goals with far greater simplicity and elegance than 2-part in situ cross-linked chemical systems.

To tune the shear-thinning properties of STHBs, several compositions were fabricated by using different concentrations of SNPs (5, 8, and 10 wt%) and PEO (1, 2, and 3 wt%) (Fig. 1b and Table S1). After formulation, the rheological properties of all STHB compositions were determined, and higher viscosity and stronger gel formation were observed on formulations with higher SNP concentrations (Fig. 1b and Table S1) [17]. For instance, the 10L3P formula presented higher viscosity (~ 727 Pa s) compared to formulations with lower SNP concentrations; 5L3P and 8L3P displayed viscosities of ~ 81 and ~ 436 Pa s when subjected to a shear of ~ 1.6 s^−1^ (Fig. 1c). A shear-thinning behavior was observed in all formulations as their viscosity decreased when shear rate was increased (Fig. S1), this conduct was expected as previous studies have shown a similar result [18].

The viscoelastic behavior of STHBs was further characterized by strain sweeps (0.001 to 1000% at 1 Hz). The linear viscoelastic region (LVR) of STHBs was obtained within a small strain region [0.001–10 strain (%) at 1 Hz], as shown in Figs. 1d and S2. Higher SNP content resulted in an increase in elastic modulus (G´) [e.g., 5L3P and 10L3P had an average of ~ 8002 and ~ 17,470 Pa at ~ 0.1 strain (%) at 1 Hz]. Posterior to the LVR [~ 10 > strain (%) at 1 Hz], a fast decrease in G´ was observed as a result of the disruption of the physical cross-linking of the gel as nanoplatelets disassembled [19]. A similar behavior was observed in the tan (δ) vs strain test, where the tan (δ) represents G”/G’; the gel point [tan (*δ*) = 1] was detected at ~ 10 strain (%), and higher elasticity was observed during a strain (%) of < 10 (tan (*δ*) < 1), with higher viscosities appearing during a strain (%) of > 10 (tan (*δ*) > 1) (Figs. 1e and S3), as reported in previous studies [20].

Low (1%) and high (100%) strains at 1 Hz were applied to STHBs over multiple cycles, demonstrating self-recovery to their original modulus (Figs. 1f and S4). The shear-thinning behavior of STHBs is caused by the ability of SNPs to disassemble and reassemble to recover its original conformation when shear stress is applied and then removed [21]. The addition of PEO (1–3 wt%) to SNPs did not affect their shear-thinning behavior, suggesting the conservation of electrostatic interactions into the nanoplatelets surface.

### STHB Can Be Effectively Administered via Injection and Spray Delivery Systems

**Fig. 2:**
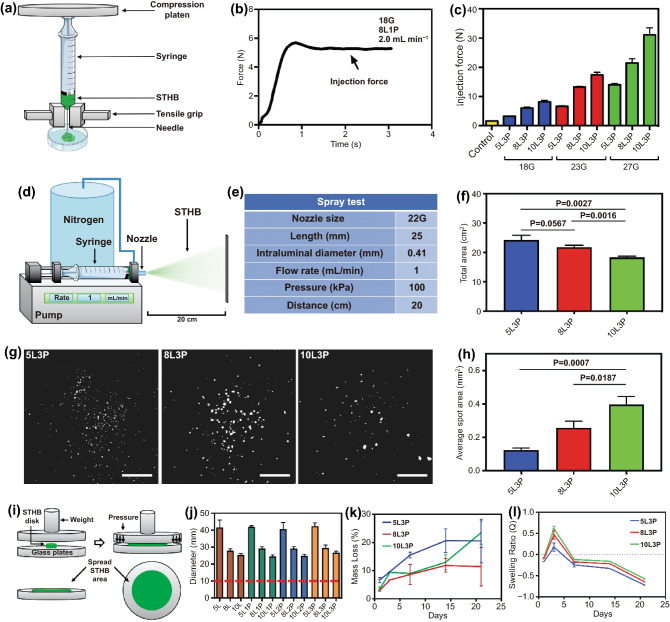
Delivery assessment and degradation kinetics of STHB. **a** Schematic representation of the setup used to characterize the injection force via a mechanical tester. **b** Measurements were performed in newtons (N), and the plateau was used to determine the maximum required injection force to extrude the hydrogels. **c** Higher SNP concentration and smaller needle intraluminal diameters resulted in higher required injection force. **d** Schematic representation of the system used to spray STHB. **e** STHB spraying was performed from a 22G nozzle, at a 1 mL/min flow rate and 100 kPa. **f** Total spray area was measured after 5 mL of STHBs were applied from a distance of 20 cm. Formulations with increased SNP concentration exhibited smaller spray areas. **g** The spot distribution of STHB formulations was captured and quantified after applying 0.1 mL of STHBs from a distance of 20 cm. Scale bars = 2 cm. **h** Average spot area was determined, and higher viscosity formulations resulted on increased average spot areas. **i** Schematic representation of the setup used to determine and measure the spreadability of STHB compositions. **j** STHB formulations with higher SNP concentration resulted in less spread area, the inclusion of PEO in the compositions did not have any effect in their spreadability. **k** Degradation kinetics of STHB formulations after 21 days. **l** Swelling ratio of STHB formulations was determined over a course of 21 days; maximum swelling occurred after 3 days of incubation. Data are represented in means ± SD, n = 3 for (**f**), (**h**), (**j**), (**k**), and (**l**), n = 5 for (**c**). *P* values were determined by Student t test (ns: *P* > 0.05)

To determine the required injection force to deliver the STHB formulations, a specialized syringe extrusion setup was mounted on a mechanical testing instrument (Figs. 2a and S5). The injectability of STHBs was evaluated on three needles with different intraluminal diameters (18G, 23G, 27G), and test parameters are presented on Table S2. The force needed to extrude the hydrogel was linearly increased until it reached a plateau defining the maximum extrusion force in each formulation (Fig. 2b). The injection force required to extrude the hydrogel compositions from the syringe was correlated to the needle size and SNP concentration. Increasing the SNP concentration (5 wt%, to 8 wt%, and 10 wt%) and decreasing the intraluminal diameter of the needle resulted in an increased extrusion force (Fig. 2c). The addition of several PEO concentrations (1, 2, and 3 wt%) to SNPs did not significantly increase the required injection force (Fig. S6). All STHBs presented an injection force well below the maximum recommended standard for injectable medical materials (~ 80 N) [22].

A spraying system equipped with a nozzle size of 22G was used to spray the 5L3P, 8L3P, and 10L3P formulations, and the average sprayed area and spot size were quantified as previously reported (Fig. 2d-e) [12]. The spray angle and total area covered from a distance of 20 cm were the following: 5L3P was 7.91 ± 0.24° and 24.28 ± 1.51 cm^2^, 7.50 ± 0.11° and 21.78 ± 0.65 cm^2^ for 8L3P and 6.88 ± 0.09° and 18.28 ± 0.46 cm^2^ for 10L3P, respectively (Fig. 2f and Table S3). Distribution and average spot area were captured and quantified by using area fraction analysis after spraying STHB formulations from a distance of 20 cm (Fig. 2g). Higher viscosity formulations had individual spots with bigger area: 0.13 ± 0.01 mm^2^ for 5L3P, 0.26 ± 0.26 mm^2^ for 8L3P and 0.40 ± 0.05 mm^2^ for 10L3P (Fig. 2h). Overall, most STHBs were injectable and sprayable (Table S4 and Videos S1-S12).

To further study STHBs topical applications, a spreading analysis was performed as previously reported [23]. The spreadability was quantified by placing hydrogels in a device where a standard weight (125 g) was applied (Fig. 2i), and classified as fluid gels, semifluid gels, semistiff gels, stiff gels, or very stiff gels based on their spreadability area (Table S5). The resulting spreading diameter of STHB compositions was in the range of 37.9 to 45.9 mm for 5 wt% SNP compositions, 26.9 to 31.4 mm for 8 wt% SNP compositions, and 23.3 to 27.5 mm for 10 wt% SNP compositions, respectively (Fig. 2j). No significant variation on spreadability was observed when PEO was included in the compositions, and formulations with higher SNP concentration resulted in less spreading area; 5 wt% SNP formulations were classified as stiff gels, and 8 wt% and 10 wt% SNP formulations were classified as gels with very high stiffness**.** After spreading the 10L3P formulation, environmental scanning electron microscopy (eSEM) was performed; micrographs show that a morphologically cohesive and robust hydrogel barrier was formed (Fig. S7). It is concluded that STHBs have sufficient mechanical properties to form spreadable barriers suitable for topical administration.

Stability in solution and swelling percentage of the most promising STHB formulations were investigated to determine their potential application as adhesion barriers. After 21 days, the mass loss percentage of STHB formulations was 20.54 ± 7.76 for 5L3P, 11.42 ± 6.86 for 8L3P, and 23.49 ± 4.12 for 10L3P (Fig. 2k). The maximum swelling ratio was 0.18 ± 0.01 for 5L3P, 0.47 ± 0.10 for 8L3P, and 0.62 ± 0.06. for 10L3P, respectively (Fig. 2l). SNP-based hydrogels are considered biodegradable materials as their exposure to pH levels below 7 accelerates their degradation, nevertheless, the observed slow degradation behavior in this hydrogel system is consistent with previous literature describing a stabilization effect of the suspension due to coverage of the nanoplatelets surface by polymers [21].

### STHB Prevents Cell Adherence While Maintaining Cell Viability in vitro

Prevention of cell adherence and infiltration to the hydrogel formulations is fundamental for the creation of an effective adhesion barrier. For this purpose, the cell–material interactions between STHBs and 3T3 fibroblasts and THP-1 monocytes were investigated.

**Fig. 3:**
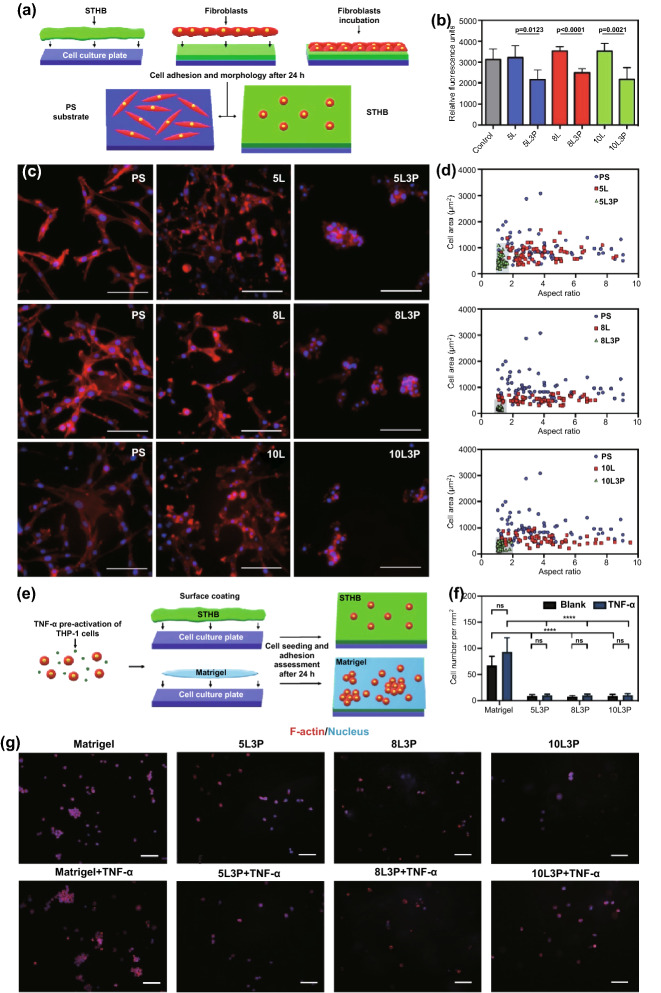
In vitro biological characterization of material–cell (STHB-fibroblast) interactions. **a** Schematic representation of the test used to determine adherence and morphological features of 3T3 fibroblasts seeded in the surface of STHB formulations. **b** 3T3 cell viability was determined by using relative fluorescence units to correlate cell numbers; the inclusion of 3 wt% PEO on STHB formulations decreased cell adherence to hydrogel surfaces significantly. **c** Representative fluorescence micrographs of cellular morphological features of 3T3 cells after 24 h of incubation on the surface of PTFE substrates (control), SNP STHB formulations (5L, 8L, 10L), and SNP/PEO STHB formulations (5L3P, 8L3P, 10L3P). In the presence of SNP/PEO STHB compositions, fibroblasts (red) exhibited limited pseudopodia expansion. Scale bars = 100 µm. **d** 3T3 cellular morphology was analyzed by quantifying the cell area and aspect ratio of the fibroblasts seeded on PTFE substrates (control), SNP STHB formulations (5L, 8L, 10L), and SNP/PEO STHB formulations (5L3P, 8L3P, 10L3P). SNP/PEO STHB compositions presented a reduced cell area and aspect ratio (gray area). The ability of THP-1 cells to adhere and infiltrate the STHB formulations was investigated after incubating the cells with the hydrogel system for 24 h; Matrigel was used as a positive control. **e** Schematic representation of the test used to determine adherence of THP-1 cells to the Matrigel matrix and STHB formulations. **f** Quantification of cell numbers per mm^2^ was performed analyzing fluorescence micrographs; it was concluded that STHB formulations provided superior efficacy in preventing cellular adhesion when compared to Matrigel. **g** Fluorescence micrographs showed that after TNF-α stimulation, THP-1 cells infiltrated and adhered to STHB formulations in significantly lower numbers when compared to Matrigel. F-actin was stained using phalloidin and can be observed in red, and nucleus was stained with DAPI and can be observed in blue. Scale bars = 100 µm. Data are represented in means ± SD, *n* = 5 for (**b**) and (**f**), *n* = 60 for (**d**). ns: not significant, **p value < 0.01, ***p value < 0.001, ****p value < 0.0001. *P* values determined by one-way analysis of variance (ANOVA). (Color figure online)

To evaluate cellular adherence, 3T3 cells were seeded on polytetrafluoroethylene (PTFE) coated substrates suitable for cell adherence (positive control) and compared to cells seeded on STHB surfaces (Fig. 3a). After 24 h, the emitted relative fluorescence units showed similar cell numbers attached to SNP-only formulations (5L, 8L, and 10L) and the control group, however, cell numbers decreased as PEO was introduced in the formulation and concentrations increased from 1 wt% to 3 wt% (Figs. 3b and S8). Formulations with 3 wt% PEO presented 32.8% (5L3P), 29.5% (8L3P), and 38.3% (10L3P) less cells, compared to 5L, 8L, and 10L. Based on these results, we determined that the most significant and efficacious formulations to prevent cell adherence were the ones containing 3 wt% PEO.

Single-cell analysis (aspect ratio and morphology) of 3T3 cells was performed via F-actin fluorescence labeling. Fluorescence micrographs showed that cells seeded on PTFE substrates (control) and SNP-only formulations (5L, 8L, 10L) had normal cell adherence and pseudopodia expansion (Fig. 3c). In contrast, when cells were seeded on STHBs with 3 wt% PEO (5L3P, 8L3P, 10L3P), the cellular morphology was spherical, as cells were unable to attach to the hydrogel surface. Previous studies have shown PEO as an efficient polymer to suppress non-specific protein adsorption and prevent cell adhesion [24].

The aspect ratio (based on dimensions, geometry, and area) of single 3T3 cells was quantified to understand cell adherence and expansion [25]. As expected, cells in control and SNP-only groups presented a disparate set of aspect ratios on the graphs shown on Fig. 3d, as individual attached cells normally differ in shape due to unique pseudopodia expansion when not in confluency [25]. Nevertheless, when cells were seeded on 5L3P, 8L3P, and 10L3P, most cells had similar aspect ratio indicating spherical morphology and low adhesion.

In the case of THP-1 monocytes, cells were pre-treated with TNF-α to activate their inflammatory phenotype 24 h before incubation in the surface of STHB formulations and Matrigel (positive control) (Fig. 3e). An untreated group of THP-1 cells was used to determine the impact of TNF-α stimulation. Quantification of cell numbers per mm^2^ confirmed a statistically significant decrease in cellular adhesion to the STHB hydrogels when compared to Matrigel matrix (Fig. 3f). As shown in the fluorescence micrographs of Fig. 3g, immune cells adhered to the Matrigel matrix in higher numbers than the STHB compositions. A similarly low number of cells was observed in the three STHB formulas. The pre-treatment of THP-1 cells with TNF-α did not appear to significantly decrease or increase cellular adhesion in any of the groups.

Biocompatibility of STHB compositions was assessed on 3T3 fibroblasts treated with SNPs, PEO, and a combination of both components (0.001 to 1000 µg mL^−1^). SNP degradation byproducts [Na^+^, Mg^2+^, Li^+^ and Si(OH)_4_] are known to be non-toxic at normal concentrations, for this purpose, a dose scalation study was performed to determine the viability limit of this nanomaterial [26]. As shown in Fig. S9a, no significant impact on cell viability was observed when SNP concentrations were increased from 0.001 to 100 µg mL^−1^, however, cell viability decreased to 70.8% at 100 µg mL^−1^, and 37.4% at 1000 µg mL^−1^. In contrast, PEO maintained cell viability with minimal toxicity at all ranges (Fig. S9b). Notably, when a mixture of SNPs and PEO was administered, no cytotoxicity was detected and excellent biocompatibility is achieved, as PEO is capable of mediating SNP-cell interactions and intracellular uptake (Fig. S9c) [27].

These results were expected as PEO has been extensively studied [28], and SNPs have demonstrated excellent biosafety across a range of cell and animal models, including degradability, unaltered viability, and anti-inflammatory effects through the release of Mg^2+^; other SNP degradation by products include Si and Li ions [9, 29, 30].

### STHB Reduces the Formation of Postoperative Adhesions in vivo

**Fig. 4:**
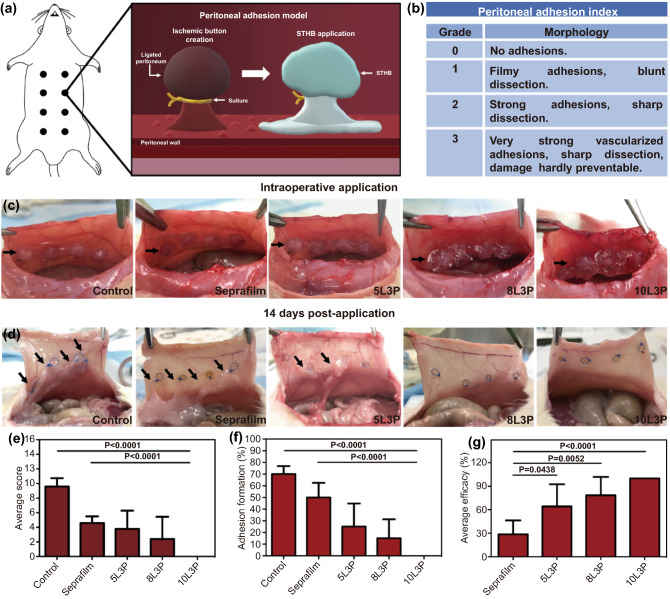
In vivo assessment of STHB efficacy in the prevention of postoperative adhesions. **a** Schematic representation of a rat peritoneal adhesion model, eight ischemic buttons were created by grasping and ligating parietal peritoneum; Seprafilm® and STHB were applied to the buttons in different groups. **b** Peritoneal adhesion index (PAI) was used to grade postoperative adhesion formation. **c** Representative images of the application of Seprafilm® and STHB formulations (5L3P, 8L3P, and 10L3P) on the peritoneal ischemic buttons, a sham group was used as a control; STHB formulations were easily delivered at the injury site achieving the formation of a resistant coating and barrier. Black arrows indicate ischemic buttons and delivery site. **d** After 14 days, the incision was reopened to evaluate adhesion formation; STHB formulations resulted in decreased adhesion formation compared to Seprafilm® and control. Black arrows indicate postoperative adhesions. **e** The average adhesion score was calculated using the PAI scoring system; 10L3P group presented the lower score. **f** The average adhesion formation (%) per rat was calculated by percentage of buttons affected; STHB formulations resulted in less amount of adhesion formations. **g** Normalized average efficacy was defined as the decrease (%) of adhesion formation compared to the control; STHB formulations presented superior performance compared to Seprafilm®. Data are represented in means ± SD, n = 5 for (**e**), (**f**), and (**g**). *P* values determined by one-way analysis of variance (ANOVA)

To investigate STHB efficacy in preventing postoperative adhesions, a peritoneal injury rat model with eight ischemic peritoneal buttons was used (Fig. 4a) [31]. Five groups were tested, a control group with no treatment (n = 5), a group administered with the commercially available adhesion barrier Seprafilm® (*n* = 5), and three groups including the most efficacious STHB formulations based on the in vitro performance results: 5L3P (*n* = 5), 8L3P (*n* = 5), and 10L3P (*n* = 5). The peritoneal adhesion index (PAI) was used as a scoring system to grade the adhesions based on several morphological features such as vascularization, thickness, strength, and damage (Fig. 4b) [32]. Surgically created ischemic buttons and application of Seprafilm®, 5L3P, 8L3P, and 10L3P can be observed on Fig. 4c. During administration, STHBs formed robust coatings that were able to properly adhere and remain in the tissue.

The formation of postoperative tissue adhesions is a pathology that develops over the course of weeks [15]. Adhesions start forming within a period of 3–7 days, and the balance between normal wound healing and excessive extracellular matrix depositions will ultimately determine the formation of adhesions at the injured site [33]. Ideally, the defect area recovers by the regrowth of mono-layered mesothelial cells in a period of 7–10 days, indicating a complete wound healing of the serosa and preventing adhesion formation [33]. Nevertheless, in several cases, postoperative adhesions are formed, and their pathophysiology occurs in several phases [34]; during the first 24 h, inflammatory infiltration and fibrin deposition occur at the injury site resulting in cytokine expression and recruiting of collagen producing cells (fibroblasts). As the injury site is covered with fibrin, the regrowth of mesothelial cells is compromised as fibroblast cells in conjunction with fibrin will start creating connective tissue (fibrotic bands) that will adhere to other organs during the first 7 days. At days 7 to 30, the gradual vascularization and collagen deposition strengthen the fibrotic bands, resulting in an undesirable tissue adhesion. Standardized animal models have described that 14 days is the best end point to evaluate the severity of adhesion formation [15, 31].

Based on this, after 14 days, animals were sacrificed, and tissues were analyzed. In the control group, adhesions were found attached to the ischemic button and its surroundings. Seprafilm®, 5L3P, and 8L3P groups had lower adhesion grades, whereas 10L3P group did not develop any observable adhesions (Fig. 4d).

To perform a quantitative analysis of the severity of adhesion formation in all the groups, parameters such as adhesion grade, number, percentage of adhesion formation, and efficacy of adhesion prevention were examined. The total average PAI score per group was 9.6 ± 0.5 for control, 4.6 ± 0.4 for Seprafilm®, 3.8 ± 2.5 for 5L3P, 2.4 ± 1.4 for 8L3P, and 0 for 10L3P (Fig. 4e). In Fig. S10, the average number of adhesions per grade for each individual group are detailed.

The average percentage of adhesion formation per group was 70% for control, 50% for Seprafilm®, 25% for 5L3P, 15% for 8L3P, and 0% for 10L3P (Fig. 4f). Based on the average percentage of adhesion formation in the control group, the normalized average efficacy preventing adhesions for the different groups was: 29% for Seprafilm®, 64% for 5L3P, 79% for 8L3P, and 100% for 10L3P (Fig. 4g). Formulations with higher viscosity presented less adhesion formation, with the most viscous composition (10L3P) providing an efficient barrier to inhibit cell infiltration and fibrotic adhesion formation.

### Tissue Remodeling and Low Immune Infiltration Following STHB Application

**Fig. 5:**
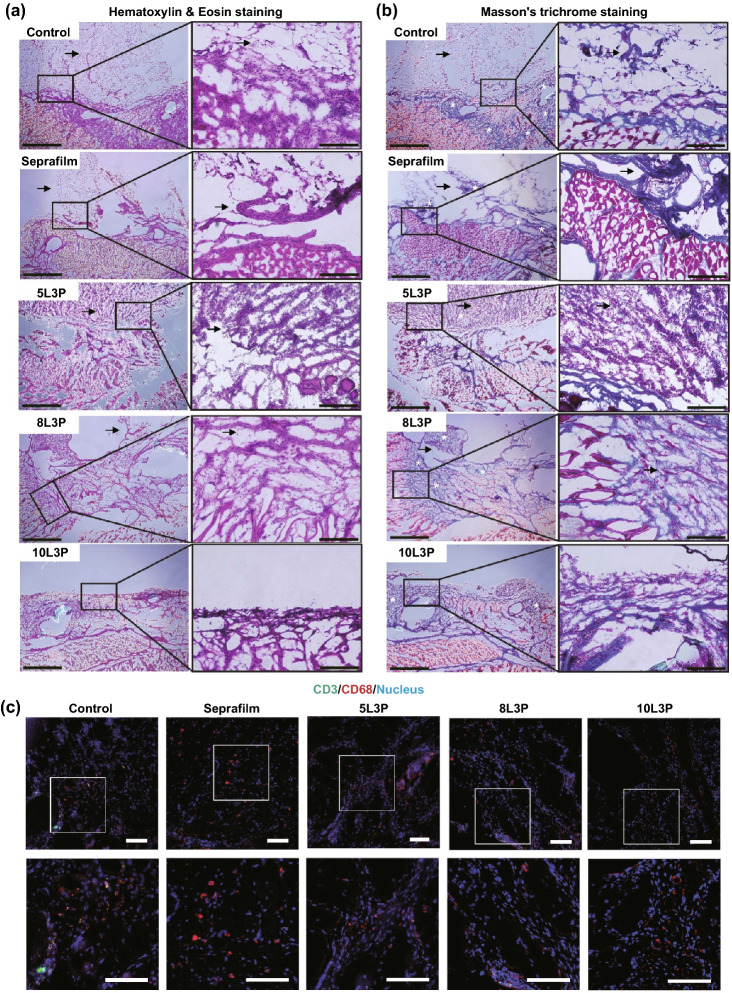
Histological examination of tissue remodeling and immune infiltration. Representative micrographs of hematoxylin and eosin (H&E) staining and Masson’s trichome staining from postoperative adhesion tissue are presented (insets represent magnified areas of the mesothelial lining, the interface where the material was applied and zones where adhesion formations could occur). **a** H&E staining was performed and the interface between the injured peritoneal lining, and fibrotic tissue can be observed in control, Seprafilm®, 5L3P, and 8L3P groups. On the 10L3P group, the peritoneal lining was preserved, and no adhesions were detected. Black arrows indicate adhesion formation areas. **b** Masson’s trichrome staining was performed in all the groups, muscular tissue can be observed in red and collagen in blue (white asterisks); highly organized fibrotic collagen bands were identified on control, Seprafilm® and 5L3P. The more viscous STHB formulations (8L3P and 10L3P) presented a decreased and more homogenous collagen distribution as observed on the micrographs and their respective magnified insets. Black arrows indicate adhesion formation areas. **c** Immunohistochemistry was performed in all the groups to determine macrophage (CD68, red) and lymphocyte (CD3, green) infiltration in response to the materials; nuclear staining (DAPI) can be observed in blue. Minimal localized immune infiltration was found in all the groups indicating negligible host immune response against the materials. Insets show magnified sections of the tissues. Scale bars on H&E (**a**) and Masson’s trichrome (**b**) micrographs are 1 mm on the left picture, and 200 µm on the magnified insets in the right. Scale bars on the fluorescence micrographs (**c**) are 100 µm for the original microscopy image and 100 µm for the insets below. (Color figure online)

After 14 days, histopathological examination was performed to evaluate tissue remodeling, inflammatory response, and STHB absorption. Hematoxylin and eosin (H&E) staining was used to evaluate morphological features of the different types of adhesions. In Fig. 5a, control group was analyzed, and a grade 3 adhesion with fibrotic bands adhered to the mesothelial lining where the ischemic button was created is presented. In the Seprafilm® group, a formation of a thin fibrotic band connected to the peritoneal wall is shown, confirming the low-grade adhesions that were observed during the PAI analysis. A grade 3 adhesion composed of a thick fibrotic band attached to the mesothelium was observed on 5L3P. 8L3P micrograph showed a grade 1 adhesion with filmy morphology and undefined interface with the peritoneal lining. Group 10L3P did not show any adhesion formation, the mesothelial lining was maintained without fibrotic band formations.

The abdominal cavity of the animal was examined, and no hydrogel or Seprafilm® barrier was found, and H&E examination confirmed its degradation in a period of 2 weeks. Rapid resorption is a key requirement for an adhesion barrier material, as lengthy residence time in the peritoneal cavity can retard re-mesothelialization of the peritoneal lining.

Masson’s trichrome staining was performed to determine the presence of fibrotic bands in the peritoneal wall (Fig. 5b). Control group micrograph shows several blue stained collagen fibrotic areas. Seprafilm® presented collagen deposition mainly in the adhesion and peritoneal interface areas. The bands found on 5L3P indicated thick collagen deposition adhered to the peritoneal lining. As compared to previous groups, 8L3P micrographs showed less dense and non-specifically distributed collagen areas consistent with local tissue remodeling. The 10L3P micrograph showed a less dense collagen deposition and a restored mesothelial lining at 2 weeks after the injury was created.

Local inflammatory response was analyzed by CD3 and CD68 immunostaining (Fig. 5c). Control and Seprafilm® groups exhibited CD3 leukocyte and CD68 macrophage infiltration as part of the wound healing process of the tissue [35]. Similarly, micrographs from STHB groups showed CD3 leukocytes and CD68 macrophages at the surgical injury site, nevertheless, compared to control and Seprafilm® groups, less inflammatory cells were observed. These observations confirmed the biocompatibility of the STHB formulations as no abnormal local immunological response was observed.

## Conclusion

In this article, we presented a novel nanoengineered hydrogel by incorporating appropriate materials with unique mechanical and biological properties that enable its injectability and sprayability and induce biological responses to prevent postoperative adhesions. The inclusion of PEO into the formulation impedes cell adherence, and in conjunction with the shear-thinning and barrier properties of SNPs, a unique and cohesive synergistic system has been fabricated.

Compared to commercially available barriers, its unique non-Newtonian properties enable its application with injectable and sprayable systems to large surface areas, such as intraperitoneal tissues by adapting to complex anatomies through the creation of a coating in their surface. These properties represent a competitive advantage with respect to reported hydrogels for the prevention of postoperative adhesions, that as a result of their chemistry (requiring cross-linking or polymerization) and high viscosity, cannot be sprayed or injected; a limiting step for their translation into clinical settings [36].

The STHB 10L3P formulation presented the best performance in preventing postoperative adhesions when compared to the other two hydrogel formulations (5L3P and 8L3P), however, all hydrogel formulas had superior efficacy than the commercially available barrier used for this study.

We conclude that the presented technology provides a novel avenue to prevent the formation of postoperative adhesions with superior efficacy, enables novel delivery methods as compared to current products in the market, and its versatility accompanied with its easy fabrication and application makes it ideal for standard and minimally invasive surgical interventions. We envision STHB as a universal platform for multiple types of surgical procedures in different areas of the body, and its translation to clinical settings will be beneficial in reducing the costs, morbidity, and mortality associated with postoperative adhesions.

## Supplementary Information

Below is the link to the electronic supplementary material.

Supplementary file1 (MP4 2520 kb)

Supplementary file2 (MP4 2515 kb)

Supplementary file3 (MP4 2506 kb)

Supplementary file4 (MP4 2523 kb)

Supplementary file5 (MP4 2521 kb)

Supplementary file6 (MP4 2523 kb)

Supplementary file7 (MP4 2560 kb)

Supplementary file8 (MP4 2531 kb)

Supplementary file9 (MP4 2586 kb)

Supplementary file10 (MP4 2525 kb)

Supplementary file11 (MP4 2525 kb)

Supplementary file12 (MP4 2530 kb)

Supplementary file13 (DOCX 7310 kb)
