# Supplementary material for: Nanoengineered Shear-Thinning Hydrogel Barrier for Preventing Postoperative Abdominal Adhesions
Source: Nanomicro Lett. 2021 Oct 18;13:212. doi: 10.1007/s40820-021-00712-5 (PMC8523737; doi:10.1007/s40820-021-00712-5)
Supplement: Supplementary file 13 — Supplementary file13 (DOCX 7310 kb) [file 40820_2021_712_MOESM13_ESM.docx]

**Supplementary information**

**Nanoengineered Shear-thinning Hydrogel Barrier**

**for Preventing Postoperative Abdominal Adhesions**

Guillermo U. Ruiz-Esparza^1,2^§, Xichi Wang^1,2,3^§, Xingcai Zhang^4^, Sofia Jimenez-Vazquez^1,2,5^, Liliana Diaz-Gomez^1,2,5^, Anne-Marie Lavoie^1,2^, Samson Afewerki^1,2^, Andres A. Fuentes-Baldemar^1,2^, Roberto Parra-Saldivar^1,2,5^, Nan Jiang^1,2,6^, Nasim Annabi^7^, Bahram Saleh^8^, Ali K. Yetisen^9^, Amir Sheikhi^1,2,10,11^ Thomas H. Jozefiak^1,2^, Su Ryon Shin^1,2^, Nianguo Dong^3^, Ali Khademhosseini^1,2,12,^*

1 Biomaterials Innovation Research Center, Division of Engineering in Medicine, Department of Medicine, Brigham and Women's Hospital, Harvard Medical School, Cambridge, MA 02139, USA

2 Division of Health Sciences and Technology, Harvard University – Massachusetts Institute of Technology, Cambridge, MA 02139, USA

3 Department of Cardiovascular Surgery, Union Hospital, Tongji Medical College, Huazhong University of Science and Technology, Wuhan 430022, China

4 School of Engineering and Applied Sciences, Harvard University, Cambridge, MA 02138, USA

5 School of Engineering and Science, and School of Medicine and Health Science, Tecnologico de Monterrey, Campus Monterrey and Campus Guadalajara, Monterrey, Nuevo Leon 64849, Mexico and Zapopan, Jalisco 45201, Mexico

6 West China School of Basic Medical Sciences and Forensic Medicine, Sichuan University, Chengdu 610041, China

7 Department of Chemical and Biomolecular Engineering, University of California, Los Angeles, Los Angeles, CA, 90095, USA.

8 Department of Chemical Engineering, Northeastern University, Boston, MA 02115, USA

9 Department of Chemical Engineering, Imperial College London, SW7 2AZ, UK

10 Department of Chemical Engineering, The Pennsylvania State University, University Park, PA 16802, USA

11 Department of Biomedical Engineering, The Pennsylvania State University, University Park, PA 16802, USA

12 Terasaki Institute for Biomedical Innovation, 11570 W Olympic Blvd, Los Angeles, CA 90024

**Address for Correspondence:**

Ali Khademhosseini, PhD.

Terasaki Institute for Biomedical Innovation, 11570 W Olympic Blvd, Los Angeles, CA 90024

Email: [khademh@terasaki.org](mailto:khademh@terasaki.org)

Phone: 1-310-206-5819

Fax: 1-310-794-5956

§ These authors contributed equally to this work.

* Corresponding Author


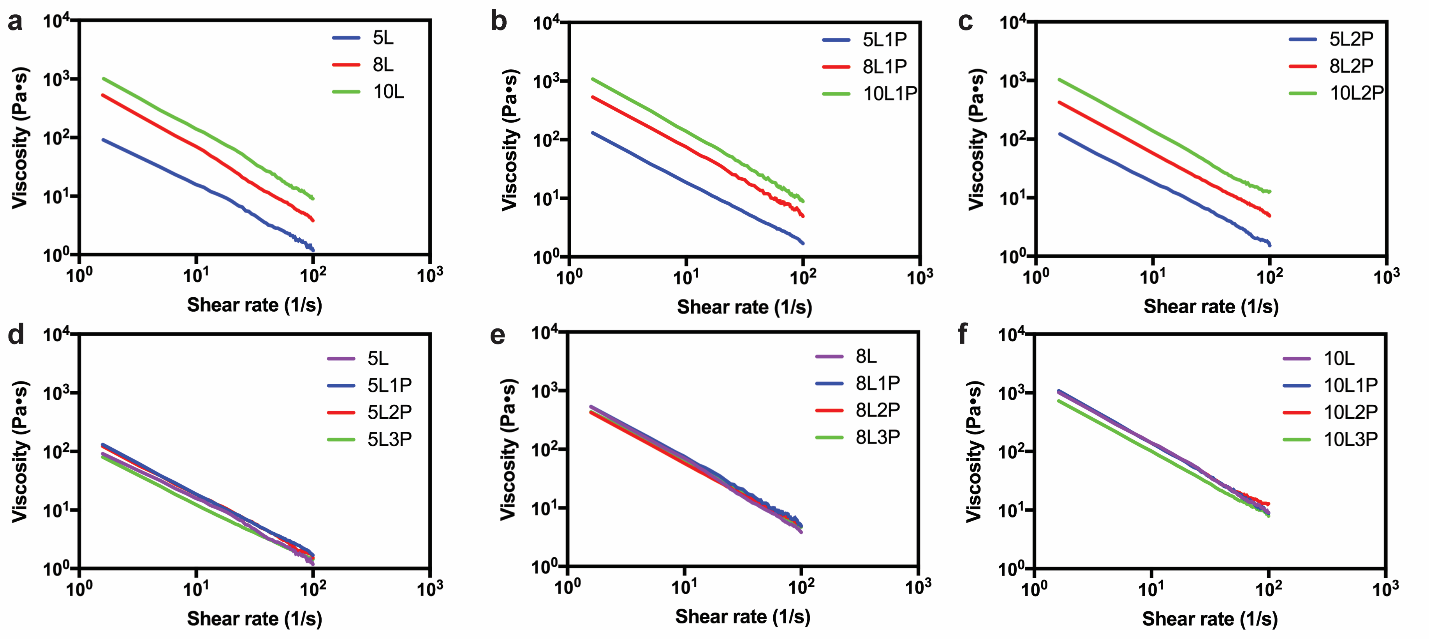


**Figure S1. Shear rate sweep assessment of STHB compositions.** The viscosity versus shear rate was characterized on STHB compositions with 0, 1, and 2 wt% PEO. **(a-c)** Rheological analysis showed that formulations with higher SNP concentrations resulted in higher viscosities and stronger gel formation. **(d-f)** The addition of 1 and 2 wt% PEO to the compositions did not impact the viscosity or shear-thinning properties, however, a slightly decrease was observed when 3 wt% PEO was introduced. In all cases, no significant effect on their shear-thinning behavior was observed.


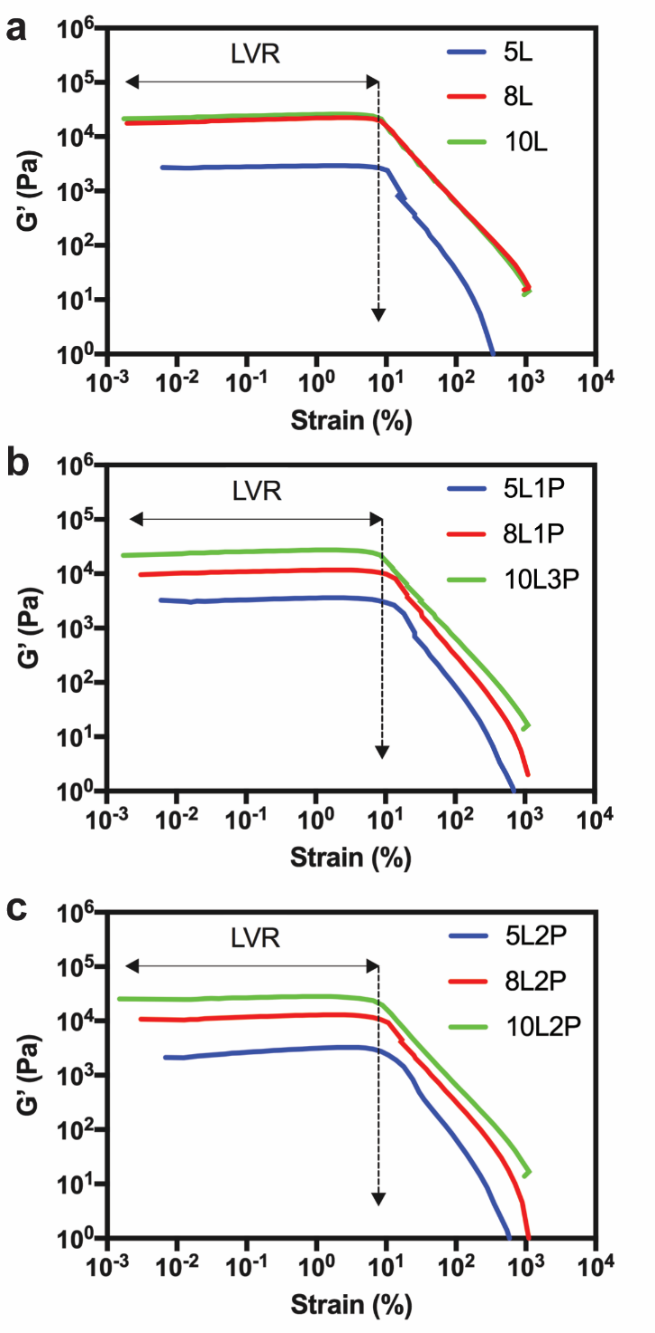


**Figure S2. Linear viscoelastic region determination on STHB compositions with 0, 1, and 2 wt% PEO. (a-c)** Strain (0.01 to 1000% at 1 Hz) versus storage modulus (G′) was quantified and the linear viscoelastic region (LVR) identified as the G’ plateau [0.1-10 strain (%)]. LVR was similar in all the compositions.


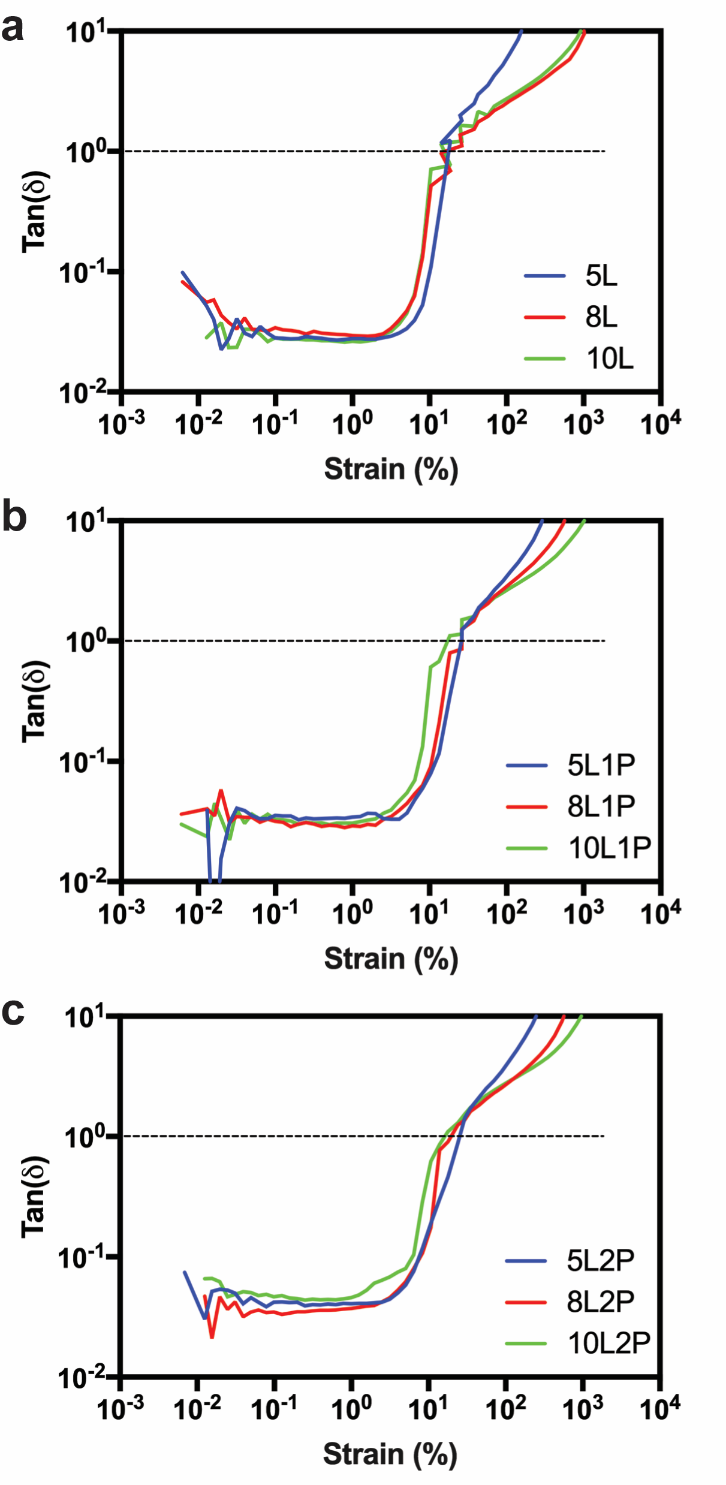


**Figure S3. Gel point of STHB compositions with 0, 1, and 2 wt% PEO.** Tan (δ) versus strain was calculated to determine the elastic to viscous transformation, and gel point defined as tan(δ) = 1. **(a-c)** The gel point of all formulations was found consistent at ~10 strain (%).


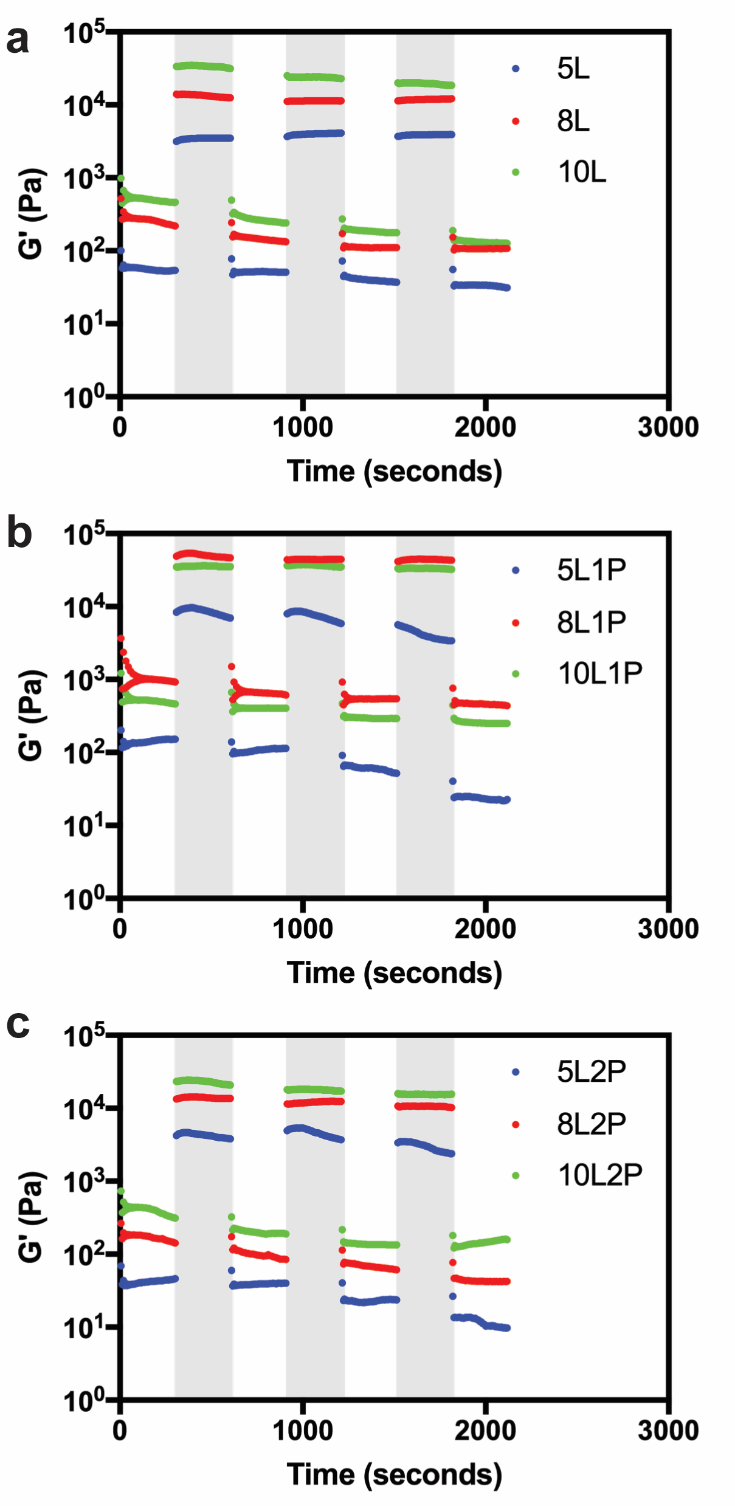


**Figure S4. Rapid mechanical recovery after applied strain on STHB compositions with 0, 1, and 2 wt% PEO.** Storage moduli (G′) was recorded during multiple cycles at low (1%) and high (100%) strain. **(a-c)** All the formulations presented rapid recovery to its original modulus (light gray regions) after strain was removed.


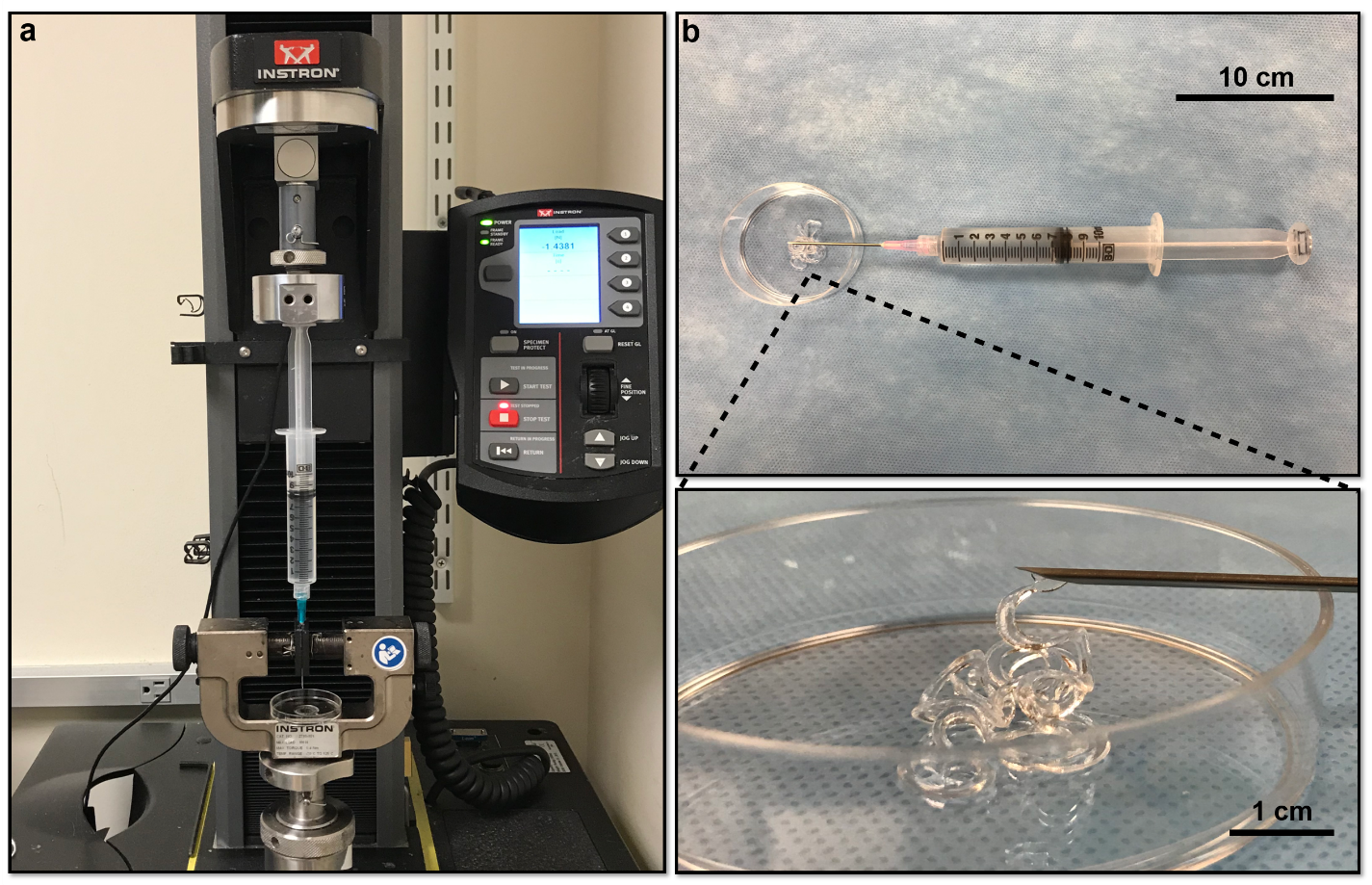


**Figure S5. STHB injection setup. (a)** Image of the set up and mechanical tester used to measure the injection force to extrude STHB formulations. **(b)** A syringe and needle were used to test the injection force of the hydrogels, STHB can be observed as a transparent compact gel.


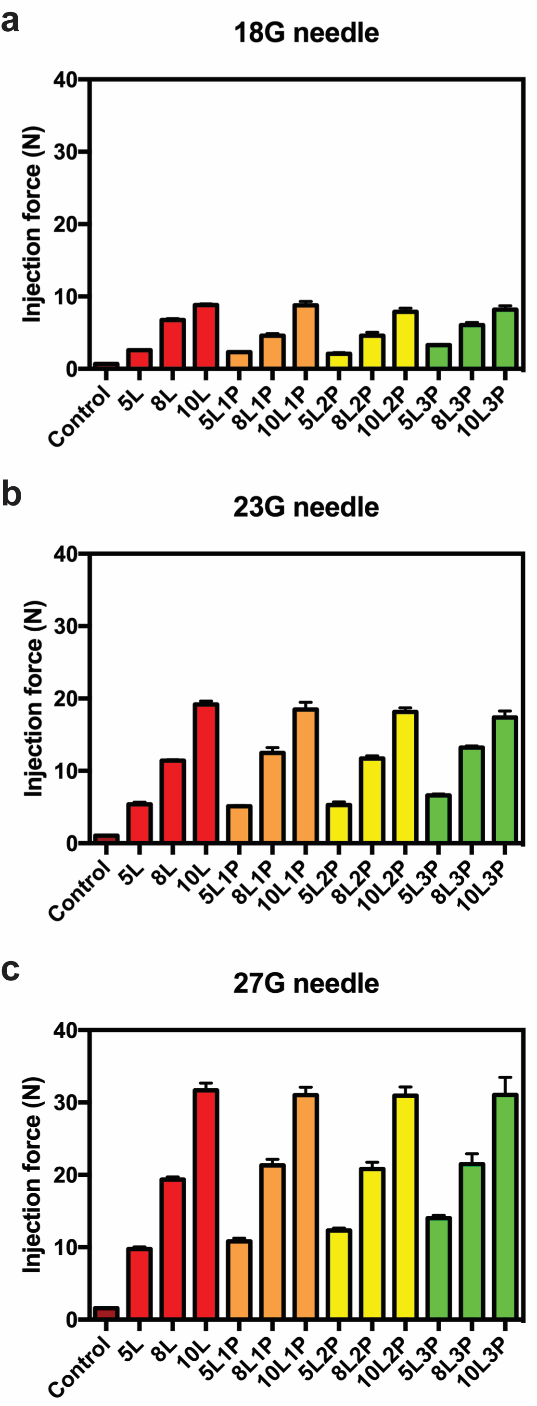


**Figure S6. STHB injection force.** Injection force of STHB formulations was measured by extruding the hydrogels through 18G **(a)**, 23G **(b)**, and 27G **(c)** needles at an infusion rate of 2 mL/min. Higher concentration of SNPs and smaller needle intraluminal diameter resulted in higher injection force; the addition of PEO did not have any significant influence on the injection force. Data is represented in mean ± SD (n=5).


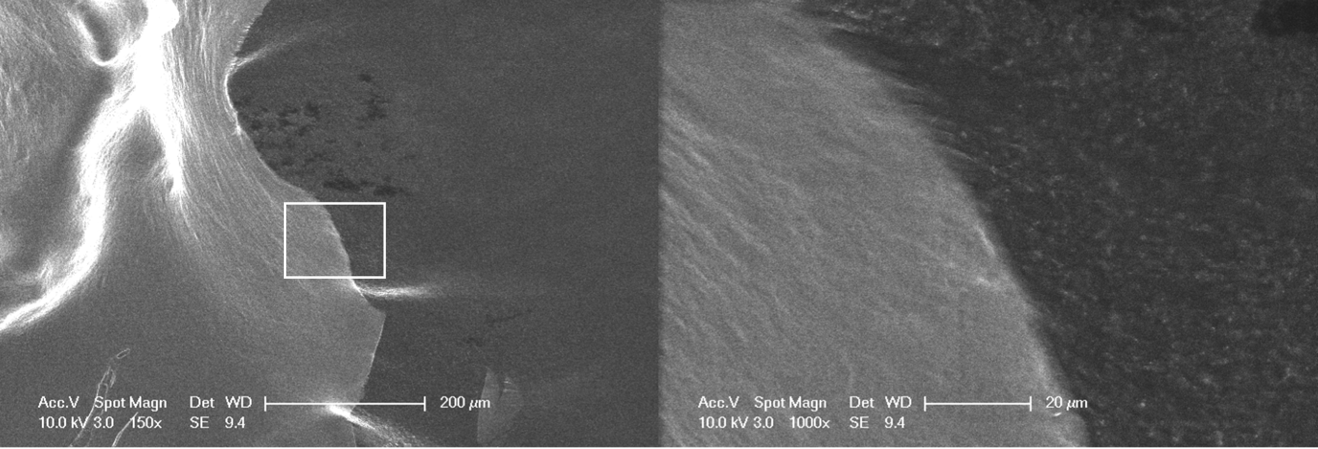


**Figure S7.** eSEM imaging showing 10L3P forming a cohesive and robust barrier with clear edges.


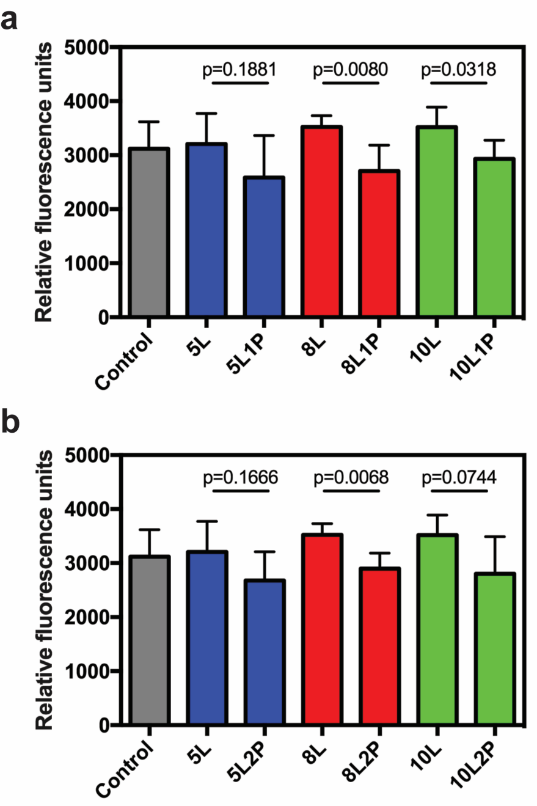
**Figure S8. Assessment of surface adherence of 3T3 cells to STHB. (a-b)** To determine the number of attached 3T3 cells, a quantification of relative fluorescence units emitted by cells seeded on the surface of STHB formulations with 0 wt% (5L, 8L, 10L), 1 wt% (5L1P, 8L1P, 10L1P) and 2 wt% (5L2P, 8L2P, 10L2P) PEO was performed, a PTFE substrate was used as a control**.** The presence of PEO in STHB formulations decreased cell adherence. Data is represented in mean ± SD (n=5). *P*-values were determined by Student t-test (ns: *P*>0.05).

**
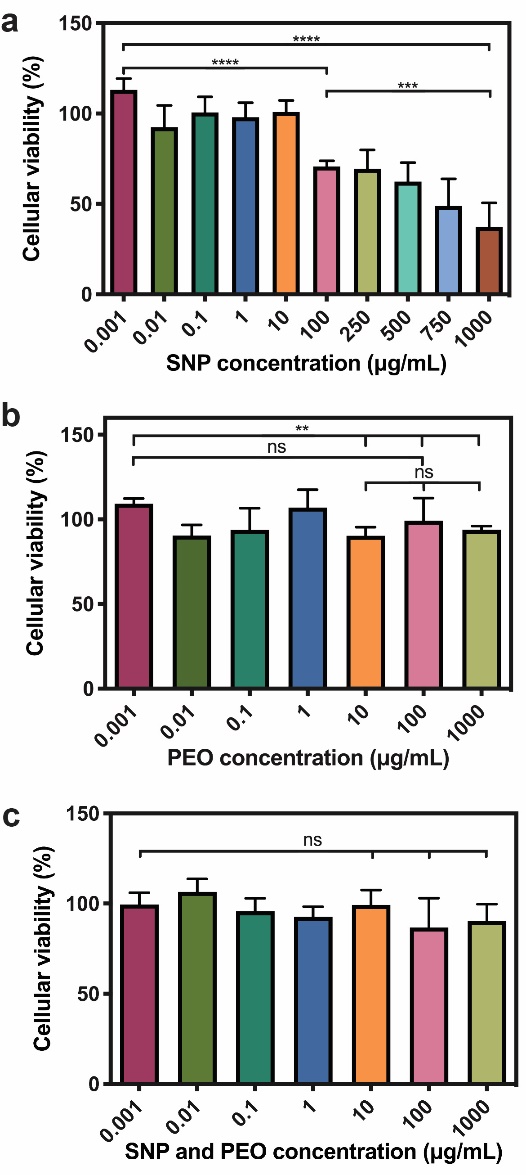
**

**Figure S9. Assessment of cellular viability to STHB components.** Cytotoxicity was evaluated in 3T3 fibroblasts after 48 hours of incubation on increasing SNP, PEO and SNP/PEO concentrations (0.001 to 1000 µg/mL). **(a)** Decreased cell viability was observed when SNP concentration was above 100 µg/Ml. **(b)** No cytotoxicity was observed when cells were exposed to PEO. **(c)** Viability was not affected when cells were exposed to a combination of SNPs and PEO. Data is represented in mean ± SD (n=5). ns: not significant, **: p-value <0.01, ***: p-value <0.001, ****: p-value <0.0001. P values determined by one-way analysis of variance (ANOVA).


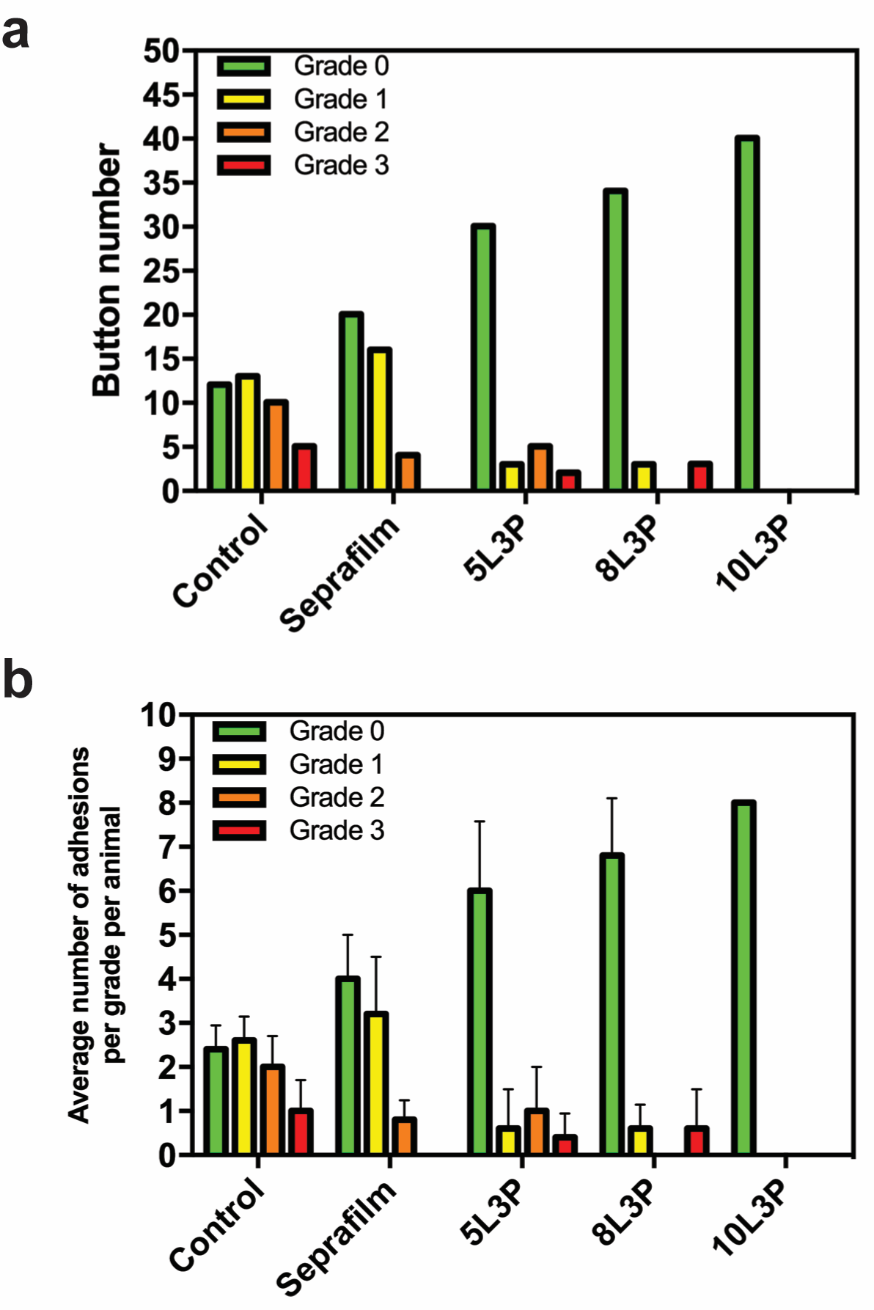


**Figure S10. Adhesion grade distribution on ischemic buttons. (a)** The total amount of adhesions by grade was quantified according to the PAI scoring system. **(b)** Average number of adhesions per grade per animal were calculated; adhesion severity was decreased with Seprafilm^®^ and STHB formulations. 10L3P was the formulation that showed superior performance with no adhesion formation. Data is represented in mean ± SD.


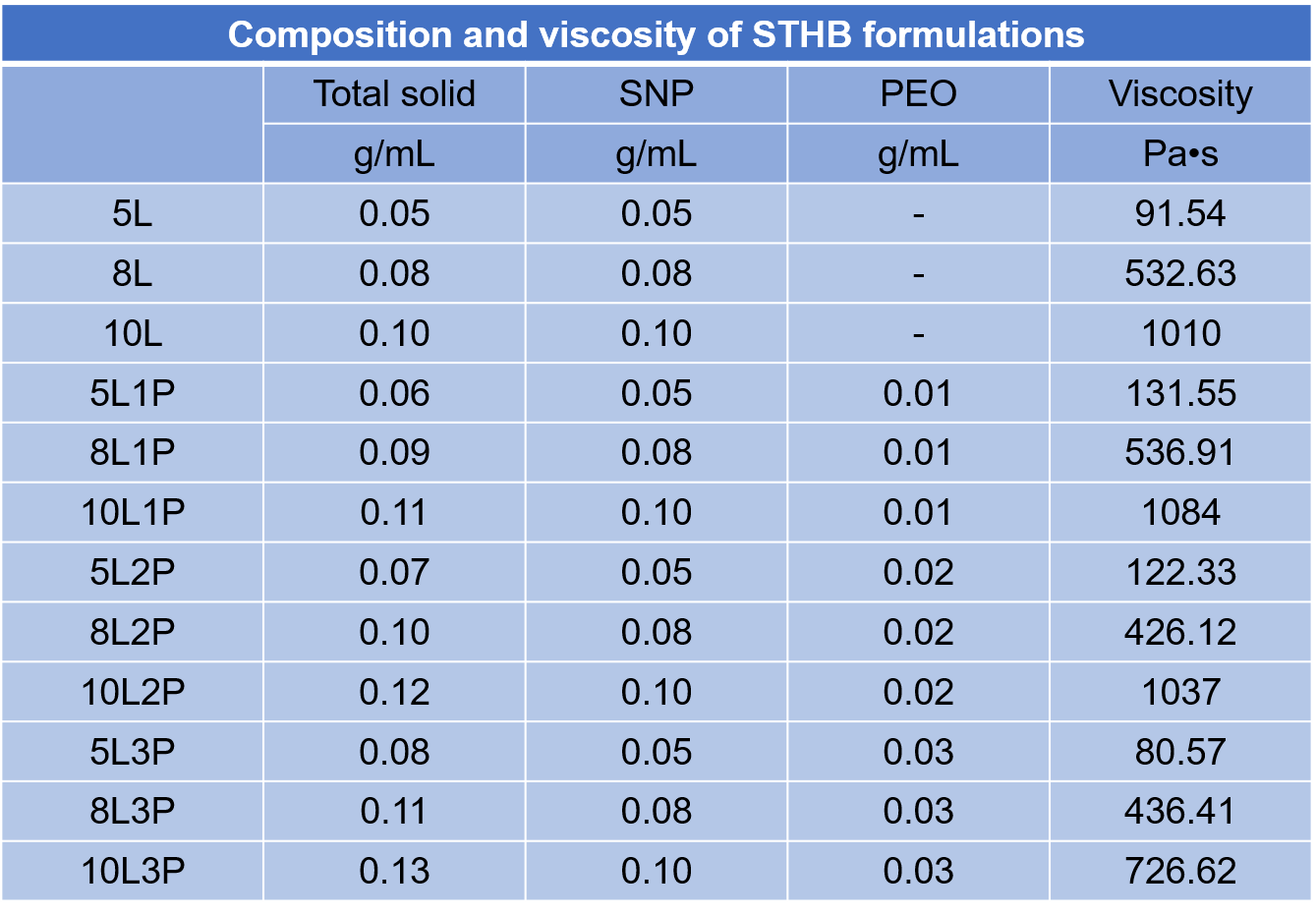


**Table S1. Composition and viscosity of STHB formulations.**


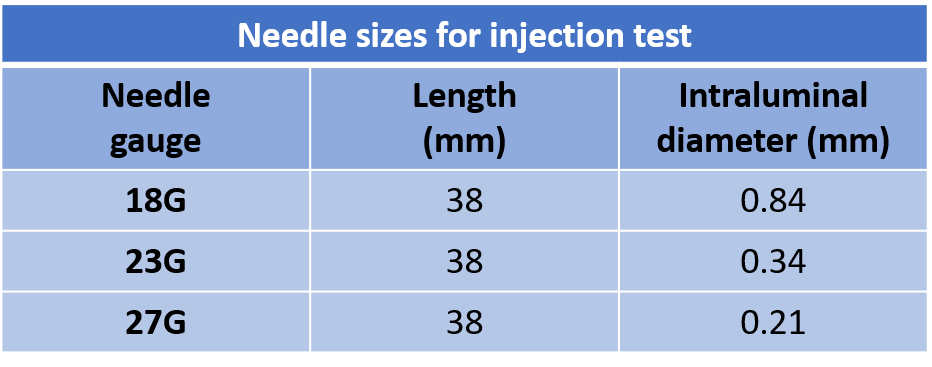


**Table S2. Needle intraluminal diameters for injection test.**

**
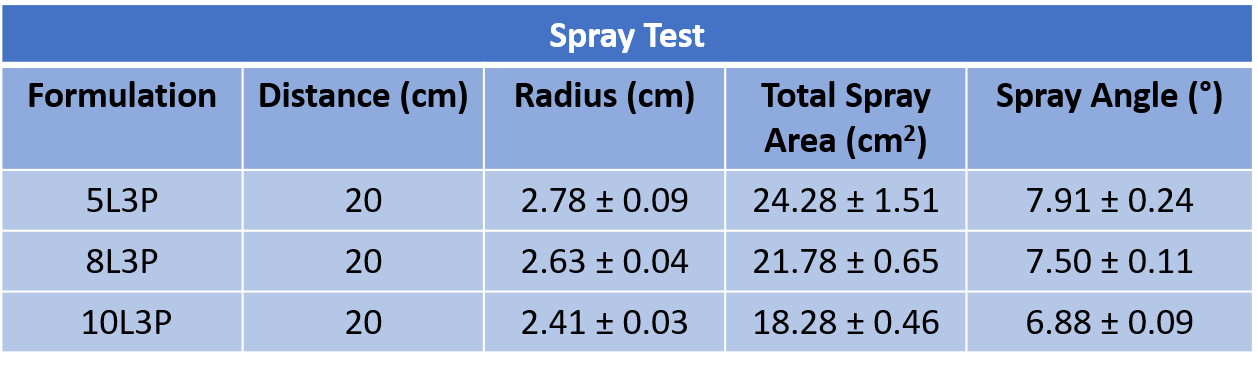
**

**Table S3. Distance, radius, total spray area and spray angle of STHB formulations.**


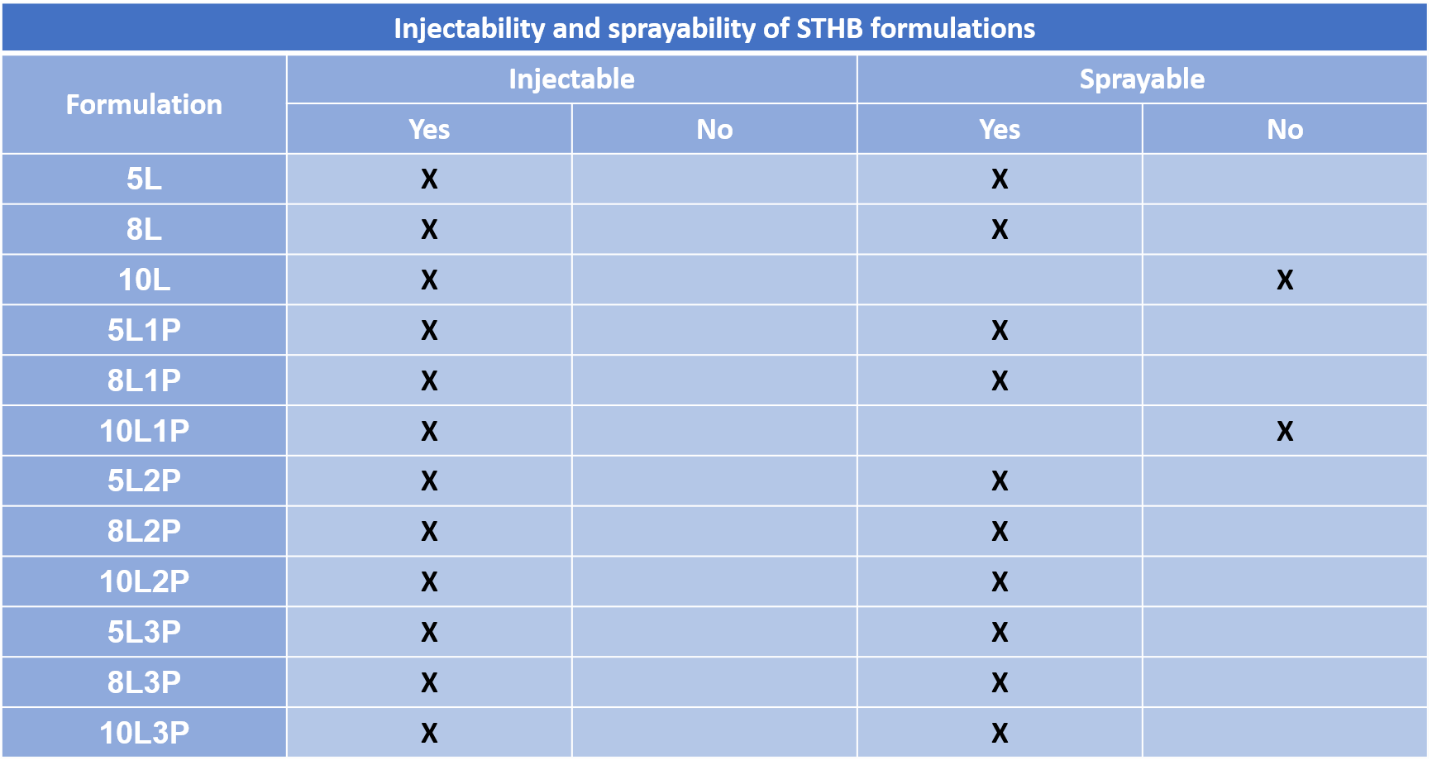


**Table S4. Injectability and sprayability of STHB formulations.**


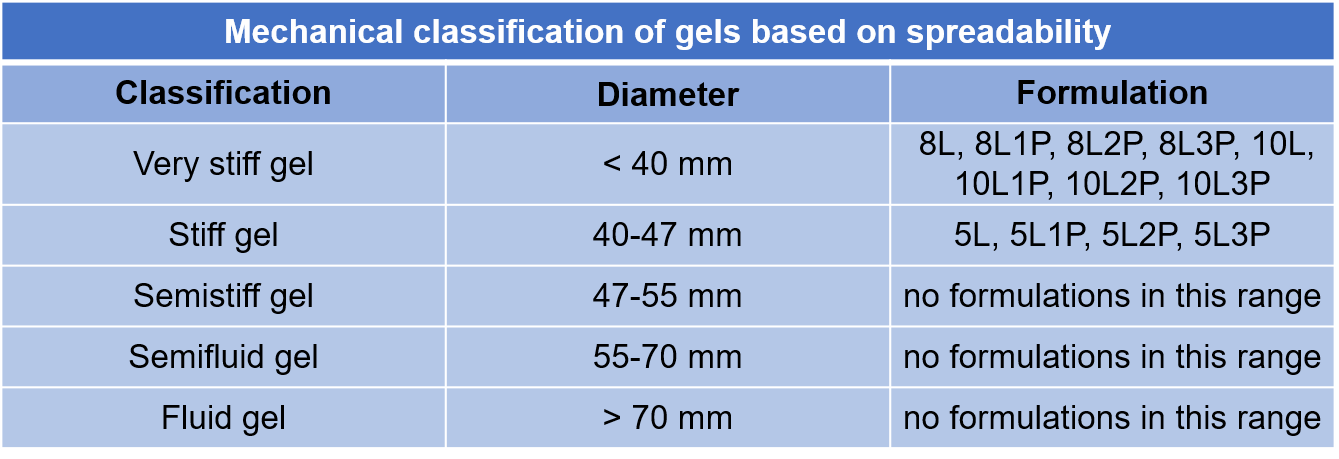


**Table S5. Mechanical classification of gels based on spreadability.**

**Supplementary Videos**

1. **Supplementary Video S1.** Spraying of 5L formulation.
2. **Supplementary Video S2.** Spraying of 8L formulation.
3. **Supplementary Video S3.** Spraying of 10L formulation.
4. **Supplementary Video S4.** Spraying of 5L1P formulation.
5. **Supplementary Video S5.** Spraying of 8L1P formulation.
6. **Supplementary Video S6.** Spraying of 10L1P formulation.
7. **Supplementary Video S7.** Spraying of 5L2P formulation.
8. **Supplementary Video S8.** Spraying of 8L2P formulation.
9. **Supplementary Video S9.** Spraying of 10L2P formulation.
10. **Supplementary Video S10.** Spraying of 5L3P formulation.
11. **Supplementary Video S11.** Spraying of 8L3P formulation.
12. **Supplementary Video S12.** Spraying of 10L3P formulation.
